# Review of 177Lu-PSMA-617 in Patients With Metastatic Castration-Resistant Prostate Cancer

**DOI:** 10.7759/cureus.8921

**Published:** 2020-06-30

**Authors:** Michael Sun, Muhammad O Niaz, Adlai Nelson, Myrto Skafida, Muhammad J Niaz

**Affiliations:** 1 Internal Medicine, Weill Cornell Medicine, New York, USA; 2 Internal Medicine, Sharif Medical City Hospital, Lahore, PAK; 3 Urology, Weill Cornell Medicine, New York, USA; 4 Radiology, Weill Cornell Medicine, New York, USA

**Keywords:** prostate cancer, prostate specific membrane antigen, radio-ligand therapy

## Abstract

Prostate-specific membrane antigen (PSMA) is a cell membrane glycoprotein that is selectively expressed in prostate cells, with expression levels increasing dramatically in prostatic adenocarcinoma. PSMA-based radioligand therapy (RLT) has emerged as a viable therapeutic modality for the treatment of progressive metastatic prostate cancer. One commonly employed combination involves lutetium-177 conjugated to the ligand PSMA-617 (^177^Lu-PSMA-617). In this meta-analysis, we examine therapeutic responses in patients with metastatic disease who have received ^177^Lu-PSMA-617 therapy. We conducted a literature search with the following inclusion criteria: clinical trials involving more than 10 patients and solely utilizing ^177^Lu-PSMA-617. Seventeen studies were included in the final analysis. Variables documented included the number of patients, the total therapeutic dose administered, the percentage of any prostate-specific antigen (PSA) decline, the percentage with PSA decline exceeding 50% baseline, and toxicities. Overall, a majority of patients responded to therapy, and in the prospective studies, survival was found to be upwards of one year. Significant toxicities included cytopenias, which were infrequent. Patients who had PSA declines in response to therapy had longer survival. Performance status and tumor grade were also key predictors of outcome.

## Introduction and background

Prostate cancer is one of the most common cancers and a major cause of mortality among men in the United States [1]. Localized prostate cancer is usually managed by surgery and radiation therapy, while androgen therapy is the mainstay of treatment for metastatic disease [2]. Metastatic castration-resistant prostate cancer (mCRPC) is defined as the clinical or biochemical progression of the disease despite the use of androgen-deprivation therapy. Therapeutic options at this stage include docetaxel, sipuleucel-T, abiraterone, and radium-223; however, chances of survival are suboptimal [3]. Thus, there is an unmet need for new therapeutic agents that can improve patient outcomes. 

Prostate-specific membrane antigen (PSMA) is a type II membrane glycoprotein, with expression drastically upregulated in prostate cancer cells. Due to its high specificity for prostate cancer, PSMA is a promising target for molecular imaging and therapeutics [4-6]. In recent years, novel imaging and therapeutic radiopharmaceuticals targeting PSMA have been developed. PSMA is internalized after binding with the radioligand, making it possible to directly deliver radiation inside the cancerous cell.

One of the promising radiopharmaceuticals that target PSMA is lutetium-177 conjugated to the ligand PSMA-617 (^177^Lu-PSMA-617). It is comprised of PSMA-617, a small molecule designed to bind with high affinity to PSMA and that target prostate cancer cells [7], the Glu-urea-Lys PSMA binding motif, and the DOTA/DOTAGA chelator linked with lutetium-177, which releases energetic beta particles that destroy cancer cells at the disease site [8,9]. ^177^Lu-PSMA-617 has an emerging role in mCRPC treatment, and there are ongoing clinical trials investigating its therapeutic responses. Herein, we present a systematic review and meta-analysis of these studies.

## Review

A literature search was conducted using PubMed and MEDLINE® search engines in February 2020. The key search terms were as follows: PSMA-617, ^177^Lu-PSMA-617, and ^177^Lutetium-PSMA-617. Each article was read in its entirety. References of included studies were also verified, and related articles not identified during regular database search were reviewed. Only those studies that met the following criteria were included: clinical trials with more than 10 patients, those with only ^177^Lu-PSMA-617, and those with documentation of prostate-specific antigen (PSA) response. Exclusion criteria included articles in languages other than English, brief communications, abstracts, letters to the editor, and case reports. All data were extracted into Microsoft Excel (Microsoft, Redmond, WA) workbook with the following information from the finalized articles: first author, year of publication, study design, baseline PSA, number of patients, the total therapeutic dose administered, PSA response (any PSA decline and PSA decline of more than 50%), and toxicities.

An electronic database search identified 200 records, and 147 records remained after duplicates were removed. Of those, 76 records were excluded due to the following reasons: being abstracts, focus on diagnostic radiotracer, concurrent use of other therapeutic radiotracers, preclinical study, or absence of radioligand therapy. After reviewing the full text of the remaining 71 articles, 54 were excluded because of focus on synthesis/dosimetry, being case reports, being reviews, or providing inadequate data. Finally, 17 studies were included in our review. The salient features of reviewed studies are summarized in Table 1.

**Table 1:** Summary of all studies included in the review PSA: prostate-specific antigen; IQR: interquartile range

No.	Study	Year	Baseline PSA, mg/ml (range)	First therapeutic dose, GBq (range)	Time of PSA evaluation	Number of patients	Any PSA decline after the first cycle	Greater than 50% PSA decline	Total number of therapy (cycle)
1	Rahbar et al. [7]	2017	214 (0.35–5436)	5.9 (2–8)	8 weeks after the first cycle	99	65/99 (66%)	40/99 (40%)	Average 1.7 (range: 1–4)
2	Ahmadzadehfar et al. [10]	2015	298.5 (5–853)	5.6 (4.1–6.1)	8 weeks after the first cycle	10	7/10 (70%)	5/10 (50%)	1
3	Ahmadzadehfar et al. [11]	2016	522 (17–2360)	6.0 (4.1–7.1)	8 weeks after the first cycle	24	19/24 (79%)	10/24 (42%)	Average 1.9 (range: 1–2)
4	Ahmadzadehfar et al. [12]	2017	510 (5–5910)	6 (4–7.2)	8 weeks after the first cycle	52	42/52 (81%)	23/52 (44%)	Average 3.6 (range: 3–6)
5	Rahbar et al. [13]	2018	361 (IQR 80–755)	6.1 (IQR 5.9–6.3)	8 weeks after the first cycle	104	70/104 (67%)	34/104 (33%)	Average 3.4 (range: 1–8), median 3
6	Hofman et al. [14]	2018	189.8 (IQR 80.1–372)	7.5 (4.4–8.7)	12 weeks after the first cycle	30	29/30 (97%)	17/30 (57%)	Median 3 (range: 2–4)
7	Maffey-Steffan et al. [15]	2019	N/A	6	8 weeks after the first cycle	32	23/32 (72%)	12/32 (38%)	2–6
8	Yadav et al. [16]	2020	333 (1.1–2493)	1.1–7.8	12 weeks after the first cycle	90	56/90 (62%)	29/90 (32%)	Median 4 (range: 1–7)
9	Yordanova et al. [17]	2019	208 (2.6–2009)	8 (6–9)	8 weeks after the first cycle	30	16/30 (53%)	7/30 (23%)	Median 3 (range: 1–6)
10	Rathke et al. [18]	2018	N/A	Stratified by 4, 6, 7.4, 9.3	8 weeks after the first cycle	40	31/40 (78%)	15/40 (38%)	3
11	Bräuer et al. [19]	2017	346 (126–881)	6.1 (IQR 5.9–6.3)	8 weeks after the first cycle	59	33/59 (56%)	13/59 (22%)	Average 2.7 (range: 1–7)
12	Rasul et al. [20]	2020	66 (1.0–4890)	7.3 ± .573	4 weeks after the third cycle	54	43/54 (80%)	31/54 (57%)	3
13	Rahbar et al. [21]	2016	342 (5–5910)	5.9 ± 0.5	8 weeks after the first cycle	74	47/74 (64%)	23/74 (31%)	1
14	Rahbar et al. [22]	2016	381 (5–1844)	5.92 ± 0.44	8 weeks after the first cycle	22	13/22 (59%)	7/22 (32%)	Average 1.8 (range: 1–2)
15	Ferdinandus et al. [23]	2017	325.5 (4.73–2360)	6.0 (4.1–7.1)	8 weeks after the first cycle	40	27/40 (68%)	13/40 (33%)	1
16	Emmett et al. [24]	2019	88 (7–2950)	7 (6–8)	Not defined	14	10/14 (71%)	5/14 (36%)	Median 3 (range: 2–4)
17	Aghdam et al. [25]	2019	217.31 (0.4–1533)	5.7 (4.4–6.6)	8 weeks after the first cycle	14	11/14 (79%)	5/14 (36%)	Median 1 (range: 1–6)

One of the earliest experiences with ^177^Lu-PSMA-617 radioligand therapy (RLT) was described by Ahmadzadehfar et al. in 2015 [10]. Ten patients with mCRPC were recruited to undergo ^177^Lu-PSMA-617 RLT and received one cycle of therapy (dose 6 GBq). At eight-week follow-up, 70% (7/10) experienced PSA decline, and 50% (5/10) experienced >50% decline in PSA levels. After those initial encouraging results, in a follow-up study with an expanded cohort, 24 patients with progressive mCRPC underwent ^177^Lu-PSMA-617 RLT [11]. After one cycle, 79.1% (19/24) showed a decline in PSA levels; 41.7% (10/24) showed >50% decline. Twenty-two out of the 24 patients were selected for a second cycle, and 68.2% (15/22) had a PSA decline, with 13 (59%) experiencing >50% decline. Since then, numerous retrospective studies with larger groups of patients have been published by the same group. In 2017, Ahmadzadehfar et al. reported a cohort of 52 patients who each received between three and six cycles of therapy (mean: 3.6 cycles, mean dose: 6 GBq) [12]. Dosing was administered in eight-week intervals and the median cumulative dose was 18.5 GBq. Of note, 80.8% (42/52) experienced a PSA decline eight weeks after the first cycle. Median overall survival was 60 weeks, with patients who had PSA responses having significantly longer survival compared to those who did not (68 vs. 33 weeks). In a cohort of 99 patients who received between one to four cycles of therapy (mean: 1.7 cycles) with 8-12 week intervals, 65.6% (45/99) had PSA response after the first cycle [7]. In a cohort of 104 patients, described in 2018, 67.3% (70/104) experienced a PSA decline after one cycle of therapy [13]. Patients underwent an average of 3.4 cycles of therapy, with eight-week intervals. The average dose was 6.1 GBq per cycle and 18.8 GBq cumulatively. The median overall survival was 56 weeks; again, those who responded had longer survival (62.9 vs. 47 weeks).

More recently, there have been several prospective studies on ^177^Lu-PSMA-617 RLT. In 2018, Hofman et al. described a group of 30 patients and noted a PSA response rate of 96.7% (29/30) [14]. Patients underwent a median of three cycles, with six weeks between each cycle. Dosing was calibrated based on weight, the extent of disease on PSMA positron emission tomography (PET) scan, and renal function; the mean dose was 7.5 GBq. Patients were excluded from the study if they had poor PSMA expression based on imaging. The median overall survival was 13.5 months. In a study involving 32 patients, also selected based on favorable PSMA expression on imaging, 71.9% (23/32) experienced PSA decline, and median overall survival was 12 months (17 months in responders vs. 11 months in non-responders) [15]. Patients underwent two to six cycles, with six-week intervals. The mean dose was 6 GBq. In the largest prospective study to date, Yadav et al. examined 90 patients with progressive mCRPC [16]. Participants underwent a median of four cycles, with eight-week intervals. The average cumulative dose was 21 GBq. Patients were selected based on PSMA PET. The median overall survival was comparable at 14 weeks, and 62.2% (56/90) had PSA declines.

With regard to predictors of response to therapy and overall survival, numerous studies have shown that patients with PSA decline in response to RLT experience longer overall survival compared to those who do not respond [12,17-25]. It also appears that the degree of PSA decline correlates with the outcome. In their study of 104 patients, Rahbar et al. found that there was no incremental benefit to overall survival beyond a PSA decline of 21% [13]. However, Rasul et al. noted that there was an added increase in survival, both progression-free and overall, with PSA declines of over 50% and over 80%, though they did not describe the extent [20]. In their prospective study, Hofman et al. showed that patients with over 50% PSA decline had longer survival compared to patients with PSA decline of under 50% (17 months vs. 9.9 months) [14].

Dosing intensity also likely correlates with overall survival and PSA response, though this association is confounded by the reality that patients with better performance status are able to tolerate higher doses and more cycles of therapy [14]. In their cohort of 104 patients, Rahbar et al. noted that patients who had received a cumulative dose exceeding 18.8 GBq had increased overall survival, even after controlling for performance status [13]. In a retrospective study, 40 patients were assigned to four different dose ranges (4, 6, 7.4, 9.3 GBq), 10 to each group; no differences in PSA decline rates were found across the groups after three cycles of therapy [18]. However, only half of the patients completed three cycles, and the majority of the patients who completed three cycles were dosed at the highest level. Furthermore, dosing intervals have varied across studies. Most have involved either six weeks or eight weeks. In a novel study, Rasul et al. retrospectively examined 54 patients who received 7.3 GBq every four weeks for three cycles [20]. Response rates were high: 79.6% (43/54) with a PSA decline, 57.4% (31/54) with over 50% decline, and 35.2% (19/54) with over 80% PSA decline. The median overall survival was 119 weeks. The authors noted that while their patient population was generally healthier than cohorts from other studies, the favorable responses and toxicity profiles associated with four-week dosing may warrant further investigation.

Regarding the predictors of poor response to RLT, it appears that poor performance status and aggressive disease are the most prominent. Several studies have identified a link between the use of opioid pain medications and lower rates of PSA decline [11,12,23]. Similarly, in a prospective study of 90 patients, multivariate regression analysis showed that higher Eastern Cooperative Oncology Group (ECOG) score was associated with worse survival (HR: 10.69) [16]. Reflecting high tumor grade, elevated alkaline phosphatase, Gleason score of 10 or higher, and the presence of visceral metastases have been reported to be negative predictors of PSA response [7,12]. It is less clear whether low PSMA expression levels result in reduced responsiveness to therapy by way of lower levels of targetable substrate. Many studies have excluded patients with poor PSMA expression on imaging, even studies that have attempted to correlate standardized uptake value (SUV) scores with PSA decline [14-16]. Consequently, studies that have shown no relationship between SUV scores and PSA decline have primarily included patients with high PSMA expression based on PET imaging.

Significant adverse events are summarized in Table 2. A minority of patients had high-grade hematological toxicities that were dose-dependent. Typically, platelets nadir around four weeks after therapy, while the leukocyte count nadirs around two weeks after therapy [18,25]. Cytopenias are generally transient, with counts eventually returning to normal ranges. Less serious, but more common, side effects included xerostomia, fatigue, and nausea [14,25].

**Table 2:** Significant adverse events reported in selected trials

Study	Toxicity
Rahbar et al. [7]	Grade 3-4 leukopenia (4 patients, 3%); grade 3-4 anemia (15 patients, 10%); grade 3-4 thrombocytopenia (5 patients, 4%); grade 3-4 fatigue (1 patient, 1%)
Ahmadzadehfar et al. [10]	Grade 4 anemia (1 patient, 10%)
Ahmadzadehfar et al. [11]	Grade 3 anemia (2 patients, 8.3%)
Hofman et al. [14]	Grade 3 lymphocytopenia (11 patients, 37%); grade 3 thrombocytopenia (3 patients, 10%); grade 4 thrombocytopenia (1 patient, 3%); grade 3 anemia (4 patients, 13%); grade 3 neutropenia (2 patients, 7%)
Yordanova et al. [17]	Grade 3 nephrotoxicity (3 patients, 10%)
Rathke et al. [18]	Grade 2 leukopenia (3 patients, 7.5%)
Rasul et al. [20]	Grade 3 leukopenia (2 patients, 3.7%); grade 3 anemia (1 patient, 1.8%)
Rahbar et al. [21]	Grade 3 anemia (1 patient, 1.4%); grade 3 thrombocytopenia (1 patient, 1.4%)
Rahbar et al. [22]	Grade 3 anemia (1 patient, 4.5%)
Ferdinandus et al. [23]	Grade 4 thrombocytopenia (1 patient, 2.5%)

## Conclusions

^177^Lu-PSMA-617 RLT has emerged as a viable and effective therapy in patients with progressive metastatic prostate cancer, with a majority of patients responding to the therapy. Most of the studies so far have been retrospective in nature, making it difficult to compare patient populations, dosing cycles, and dosing intensities. Moreover, healthier patients have been likely able to tolerate higher doses, with more frequent dosing schedules. Not surprisingly, patients with better performance status and less aggressive disease have had better outcomes. Patients who had biochemical responses to therapy have had longer overall survival compared to those who did not. Significant toxicities have included cytopenias, which are generally transient.

## References

[1] Siegel RL, Miller KD, Jemal A (2020). Cancer statistics, 2020. CA Cancer J Clin.

[2] Michaelson MD, Cotter SE, Gargollo PC, Zietman AL, Dahl DM, Smith MR (2008). Management of complications of prostate cancer treatment. CA Cancer J Clin.

[3] Crawford ED, Higano CS, Shore ND, Hussain M, Petrylak DP (2015). Treating patients with metastatic castration resistant prostate cancer: a comprehensive review of available therapies. J Urol.

[4] Silver DA, Pellicer I, Fair WR, Heston WD, Cordon-Cardo C (1997). Prostate-specific membrane antigen expression in normal and malignant human tissues. Clin Cancer Res.

[5] Wang X, Yin L, Rao P, Stein R, Harsch KM, Lee Z, Heston WD (2007). Targeted treatment of prostate cancer. J Cell Biochem.

[6] Santoni M, Scarpelli M, Mazzucchelli R, Lopez-Beltran A, Cheng L, Cascinu S, Montironi R (2014). Targeting prostate-specific membrane antigen for personalized therapies in prostate cancer: morphologic and molecular backgrounds and future promises. J Biol Regul Homeost Agents.

[7] Rahbar K, Ahmadzadehfar H, Kratochwil C (2017). German multicenter study investigating 177Lu-PSMA-617 radioligand therapy in advanced prostate cancer patients. J Nucl Med.

[8] Lorenzoni A, Capozza A, Seregni E, Giovanella L (2019). Nuclear medicine theranostics: between atoms and patients. Nuclear Medicine Therapy.

[9] Ruigrok EAM, van Weerden WM, Nonnekens J, de Jong M (2019). The future of PSMA-targeted radionuclide therapy: an overview of recent preclinical research. Pharmaceutics.

[10] Ahmadzadehfar H, Rahbar K, Kürpig S (2015). Early side effects and first results of radioligand therapy with (177)Lu-DKFZ-617 PSMA of castrate-resistant metastatic prostate cancer: a two-center study. EJNMMI Res.

[11] Ahmadzadehfar H, Eppard E, Kürpig S (2016). Therapeutic response and side effects of repeated radioligand therapy with 177Lu-PSMA-DKFZ-617 of castrate-resistant metastatic prostate cancer. Oncotarget.

[12] Ahmadzadehfar H, Wegen S, Yordanova A (2017). Overall survival and response pattern of castration-resistant metastatic prostate cancer to multiple cycles of radioligand therapy using [177Lu] Lu-PSMA-617. Eur J Nucl Med Mol Imaging.

[13] Rahbar K, Boegemann M, Yordanova A, Eveslage M, Schäfers M, Essler M, Ahmadzadehfar H (2018). PSMA targeted radioligandtherapy in metastatic castration resistant prostate cancer after chemotherapy, abiraterone and/or enzalutamide. A retrospective analysis of overall survival. Eur J Nucl Med Mol Imaging.

[14] Hofman MS, Violet J, Hicks RJ (2018). [177Lu]-PSMA-617 radionuclide treatment in patients with metastatic castration-resistant prostate cancer (LuPSMA trial): a single-centre, single-arm, phase 2 study. Lancet Oncol.

[15] Maffey-Steffan J, Scarpa L, Svirydenka A (2020). The 68 Ga/177 Lu-theragnostic concept in PSMA-targeting of metastatic castration-resistant prostate cancer: impact of post-therapeutic whole-body scintigraphy in the follow-up. Eur J Nucl Med Mol Imaging.

[16] Yadav MP, Ballal S, Bal C, Sahoo RK, Damle NA, Tripathi M, Seth A (2020). Efficacy and safety of 177Lu-PSMA-617 radioligand therapy in metastatic castration-resistant prostate cancer patients. Clin Nucl Med.

[17] Yordanova A, Linden P, Hauser S (2019). Outcome and safety of rechallenge [177 Lu] Lu-PSMA-617 in patients with metastatic prostate cancer. Eur J Nucl Med Mol Imaging.

[18] Rathke H, Giesel FL, Flechsig P (2018). Repeated 177Lu-labeled PSMA-617 radioligand therapy using treatment activities of up to 9.3 GBq. J Nucl Med.

[19] Bräuer A, Grubert LS, Roll W, Schrader AJ, Schäfers M, Bögemann M, Rahbar K (2017). 177 Lu-PSMA-617 radioligand therapy and outcome in patients with metastasized castration-resistant prostate cancer. Eur J Nucl Med Mol Imaging.

[20] Rasul S, Hacker M, Kretschmer-Chott E (2020). Clinical outcome of standardized 177 Lu-PSMA-617 therapy in metastatic prostate cancer patients receiving 7400 MBq every 4 weeks. Eur J Nucl Med Mol Imaging.

[21] Rahbar K, Schmidt M, Heinzel A (2016). Response and tolerability of a single dose of 177Lu-PSMA-617 in patients with metastatic castration-resistant prostate cancer: a multicenter retrospective analysis. J Nucl Med.

[22] Rahbar K, Bode A, Weckesser M, Avramovic N, Claesener M, Stegger L, Bögemann M (2016). Radioligand therapy with 177Lu-PSMA-617 as a novel therapeutic option in patients with metastatic castration resistant prostate cancer. Clin Nucl Med.

[23] Ferdinandus J, Eppard E, Gaertner FC (2017). Predictors of response to radioligand therapy of metastatic castrate-resistant prostate cancer with 177Lu-PSMA-617. J Nucl Med.

[24] Emmett L, Crumbaker M, Ho B (2019). Results of a prospective phase 2 pilot trial of 177Lu-PSMA-617 therapy for metastatic castration-resistant prostate cancer including imaging predictors of treatment response and patterns of progression. Clin Genitourin Cancer.

[25] Aghdam RA, Amoui M, Ghodsirad M (2019). Efficacy and safety of 177Lutetium-prostate-specific membrane antigen therapy in metastatic castration-resistant prostate cancer patients: first experience in West Asia-a prospective study. World J Nucl Med.

